# The CHROME Study, a Real-world Experience of Single- and Multiple-Dose Oritavancin for Treatment of Gram-Positive Infections

**DOI:** 10.1093/ofid/ofz479

**Published:** 2019-11-04

**Authors:** Mark Redell, Miguel Sierra-Hoffman, Maha Assi, Markian Bochan, David Chansolme, Anurag Gandhi, Kathleen Sheridan, Ivan Soosaipillai, Thomas Walsh, Jill Massey

**Affiliations:** 1 Melinta Therapeutics, Morristown, New Jersey, USA; 2 Director of Research, Texas A&M affiliated Detar Family Medicine Program & College of Medicine, Victoria, Texas, USA; 3 Texas A&M University, Bryan, Texas, USA; 4 IDC Clinical Research, Wichita, Kansas, USA; 5 Infectious Diseases of Indiana, Indianapolis, Indiana, USA; 6 Infectious Disease Consultants of Oklahoma City, Oklahoma City, Oklahoma, USA; 7 Birmingham Infectious Disease and Infusion, Birmingham, Alabama, USA; 8 University of Pittsburgh Physicians, Pittsburgh, Pennsylvania, USA; 9 Infectious Disease Associates of North Central Florida, Ocala, Florida, USA; 10 Allegheny Health Network, Pittsburgh, Pennsylvania, USA

**Keywords:** ABSSSI, oritavancin, skin infections, registry, real-world experience

## Abstract

**Background:**

Oritavancin (ORI) is a long-acting lipoglycopeptide indicated for the treatment of adult patients with acute bacterial skin and skin structure infections (ABSSSIs) caused or suspected to be caused by susceptible Gram-positive (GP) pathogens.

**Methods:**

Data collected from a retrospective observational program (2014–2017), Clinical and Historic Registry and Orbactiv Medical Evaluation (CHROME), describe the utilization, outcomes, and adverse events (AEs) associated with ORI in 440 patients treated at 26 US sites for ABSSSI and other GP infections.

**Results:**

Clinical success in evaluable patients receiving at least 1 dose of oritavancin was 88.1% (386/438). In a subgroup of patients who received ORI for skin and soft tissue infections (n = 401) and bacteremia (n = 7), clinical success was achieved in 89.0% and 100%, respectively. A cohort of 32 patients received 2–10 ORI doses separated by no more than 14 days for complicated GP infections. Clinical success was observed in 30 of 32 patients (93.8%), including 10 of 11 (90.9%) patients with bone and joint infections and 7 of 8 (87.5%) patients with osteomyelitis. In the safety evaluable population, the overall rate of AEs was 6.6%.

**Conclusions:**

We describe results from a real-world program that includes the largest multicenter, retrospective, observational study in patients who received at least 1 dose of ORI for the treatment of GP infections. This study confirms that ORI is an effective, well-tolerated antibiotic used in single and multiple doses for the treatment of ABSSSIs and complicated GP infections.

In attempts to shorten or avoid hospitalization without increasing overall health care costs, 2 long-acting lipoglycopeptide molecules, oritavancin and dalbavancin, have been approved for use in acute bacterial skin and skin structure infections (ABSSSIs). Both agents have very long half-lives, >200 hours, and are active against methicillin-resistant *Staphylococcus aureus* (MRSA) and various other skin organisms. Clinical study results for both agents have shown similar efficacy and safety as vancomycin at key end points in the treatment of ABSSSIs [1].

Oritavancin (Orbactiv; Melinta Therapeutics, Morristown, NJ, USA) is a bactericidal antibiotic that is approved in the United States for the treatment of adult patients with ABSSSI caused by designated Gram-positive pathogens including MRSA [2]. Two identical phase 3, international, randomized, and double-blind trials, SOLO I and SOLO II, demonstrated that a single 1200-mg intravenous dose of oritavancin infused over 3 hours was noninferior to vancomycin at a dose of 1 g or 15 mg/kg every 12 hours for 7–10 days for the treatment of patients with ABSSSI [3–5]. Oritavancin was introduced to the US market in late 2014. Intravenous dalbavancin (Dalvance; Allergan USA, Madison, NJ, USA) was initially studied using a 2-dose regimen given 7 days apart. Subsequently, to eliminate the need for patients not returning for the second infusion, a single dose of dalbavancin was studied and has been shown to be noninferior to 2 doses of the drug [6, 7].

Several studies of both dalbavancin and oritavancin have been reported, predominantly in skin infections, but have included complicated infections such as prosthetic joint and osteomyelitis [8]. The use of these agents for longer courses of therapy would not only potentially decrease hospital admissions, but also prevent the need for prolonged intravenous catheterization, which in turn could decrease line complications.

The consistently demonstrated safety and efficacy of oritavancin used for ABSSSI have stimulated some clinical experimentation with multiple-dose regimens for treatment of complicated and deep-seated Gram-positive infections. Several patient cases and case series describe the use of oritavancin in multiple-dose regimens for the treatment of bone and joint infections, pneumonia, bacteremia, and complicated surgical site infections [9–16]. This report characterizes the results from 2 retrospective, sequential, and observational phases of CHROME in a total of 440 patients who received oritavancin. The first phase has been reported [17].

## METHODS

### Study Design and Patient Population

Patients who received at least 1 dose of oritavancin were included in the study. Each site enrolled at least 15 consecutive patients between October 2014 and October 2017. Data collection procedures are described elsewhere [17] and emphasized below where relevant.

### Inclusion and Exclusion Criteria

Patients who received oritavancin were identified from the institution’s electronic medical records or other database and could be included regardless of infection type or previous or concomitant use of other antibiotics. To be included in the study, patients had to (1) be treated with oritavancin for a suspected or confirmed Gram-positive infection and (2) have received the last dose of oritavancin at least 60 days before data entry into the electronic case report form (eCRF). A patient was not eligible for retrospective data collection if the patient received oritavancin as part of a controlled clinical trial or as part of a sponsored pharmacoeconomic outcomes study. Waivers of informed consent were obtained from institutional review boards overseeing participating sites given the retrospective nature of the study and de-identification of patient information collected through the data entry process and final aggregation of data.

### Safety Assessments and Reporting

Safety definitions were established before patient enrollment and were included in the study protocol. Safety data were collected up to 60 days after the last dose of oritavancin. Adverse events with a reasonable possibility of a causal relationship to oritavancin, as assessed by the investigator, were reported and categorized based on their seriousness and severity. Serious adverse events (SAEs) were defined as events that resulted in death, were life-threatening, resulted in persistent or significant disability or incapacity, required prolonged hospitalization, or were medically significant events that may have jeopardized the patient or required medical or surgical intervention to prevent 1 of the previously listed outcomes. SAEs, and seriousness and severity of AEs, were collected for regulatory reporting. All SAEs, adverse events of special interest (AESIs), and pregnancies within 60 days of oritavancin infusion were reported by the investigator within 24 hours of discovery.

### Data Collection Form and Process

Investigators were trained on the use of a standardized eCRF instrument. Sites utilized eClinicalOS (IBM Clinical Development, Durham, NC, USA) as the data entry platform. Site audits were conducted remotely through a series of validation steps and data queries.

### Clinical and Microbiologic Assessments

Clinical categories of efficacy were assessed from the end of infusion of the last dose through 30 days. Clinical categories of efficacy were defined as 1 of the following: clinical cure (clinical signs and symptoms resolved), clinical improvement (partial resolution of clinical signs and symptoms), clinical failure (inadequate resolution, new or worsening clinical signs and symptoms, or need for additional nonoritavancin therapy for treatment of the baseline infection), or nonevaluable. Clinical assessments included several measures of the primary Gram-positive infection and included vital signs and white blood cell count (both incorporated into the definition of systemic inflammatory response syndrome [SIRS]) (Table 1) and cessation of spreading or reduction in the size of the baseline lesion. Patient outcomes were classified as cure or improvement in the presence of a positive culture of the baseline pathogen at end of therapy if the investigator determined that persistence was a result of colonization and no additional antibiotic therapy was instituted.

**Table 1.** Demographics and Baseline Characteristics for Oritavancin-Treated Patients

Characteristic	Value or No. (%)
Age, y	
Mean (SD)	57.8 (16.4)
Median (IQR)	58.7 (46.8–69.6)
Range	18–98
≥65 y	37.0
Male, %	53.2
Race, white, %	93.0
BMI, kg/m^2^	
Mean (SD)	32.8 (9.0)
Median (IQR)	31.4 (26.2–38.0)
Range	14–65
SIRS at presentation, No. (%)^a^	37 (8.3)
Temperature >38ºC, No. (%)	9 (2.0)
WBC > 12 000 cells/mm^3^, No. (%)	38 (15.6)
Comorbidities, No. (%)	
Hypertension	235 (53.4)
Diabetes mellitus	174 (39.5)
Diabetic neuropathy	54/174 (31.0)
Diabetic foot infection	38/174 (21.8)
Hyperlipidemia	138 (31.4)
Peripheral vascular disease/lymphedema	76 (17.3)
Coronary artery disease	63 (14.3)
COPD/asthma	58 (13.2)
Chronic kidney disease	41 (9.3)
Neoplastic disease	31 (7.0)

Abbreviations: BMI, body mass index; COPD, chronic obstructive pulmonary disease; IQR, interquartile range; SIRS, systemic inflammatory response syndrome; WBC, white blood cell count.

^a^Systemic inflammatory response syndrome is defined as 2 of the following: temperature >38°C, pulse >90 beats per minute, respiratory rate >20 breaths per minute, white blood cell count >12 000 mm^3^ or <4000 mm^3^, or >10% bandemia.

Microbiological response categories for assessments conducted between the end of infusion and 30 days after the dose of oritavancin considered only Gram-positive pathogens believed to be related to the primary infection process. Microbiological response was defined as either microbiologic eradication (documentation of a negative bacterial culture from the same site as the initial positive baseline culture) or microbiologic persistence (bacterial growth of the same organism from the same site as the initial positive baseline culture). Patients were not required to have post-therapy cultures to evaluate eradication or persistence, and these were performed as clinically indicated by the investigator.

### Statistical Analysis

This was a descriptive study of patients infected with Gram-positive pathogens.

## RESULTS

### Patient Population and Demographics/Baseline Characteristics

Data for 440 patients were collected from 26 geographically dispersed US health care sites. Patient demographics and baseline characteristics are presented in Table 1. Oritavancin was administered to a physiologically and medically heterogeneous population; obesity and significant baseline medical conditions, such as hypertension, diabetes, and hyperlipidemia, were prevalent. Despite multiple comorbidities in this population (ie, hypertension, diabetes, hyperlipidemia), these patients were not seriously ill considering infrequent baseline characteristics such as SIRS or elevated white blood cell count.

### Infection Classification and Microbiology

Among 440 patients with identified infection type listed in Table 2, skin and soft tissue infections (SSTIs) accounted for the majority of infections (91.1%), and cellulitis was the most prevalent infection type (67.3%). Wound infections (surgical, traumatic, and other) and abscesses followed, with prevalence rates of 16.7% and 16.0%, respectively. Seven patients received oritavancin for bacteremia; these were documented microbiologically as MRSA (2), methicillin-susceptible *S. aureus* (MSSA) (1), *Staphylococcus epidermidis* (2), and unspeciated Gram-positive bacteria (2). The remaining 32 (7.3%) patients had other infections and, for at least 2 patients, included with osteomyelitis as 18 patients, septic arthritis/synovitis (4), prosthetic joint infection (3), and infected bursa (3).

**Table 2.** Infection Classification, Microbiology and Prior Antibiotic Use

Infection Classification	no./No. (%)
Skin and soft tissue	401/440 (91.1)
Cellulitis	270/401 (67.3)
Wound	67/401 (16.7)
Abscess	64/401 (16.0)
Bacteremia	7/440 (1.6)
Primary	5/7 (71.4)
Secondary to SSTI	2/7 (28.6)
Other	32/440 (7.3)
Osteomyelitis	18/32 (56.3)
Septic arthritis/synovitis	4/32 (12.5)
Prosthetic joint infection	3/32 (9.4)
Infected bursa	3/32 (9.4)
Catheter exit site	1/32 (3.1)
Maxillary sinus infection	1/32 (3.1)
Hardware, posterior lumbar tissue	1/32 (3.1)
Lymphadenitis	1/32 (3.1)
Gram-positive cultures	146/440 (33.2)
*Staphylococcus aureus*	108/146 (74.0)
MRSA	64/108 (59.3)
MSSA	44/108 (40.7)
Streptococci (groups A, B, viridans)	15/146 (10.3)
Prior antibiotic therapy^a^	314/440 (71.4)
Cephalosporins^b^	154/314 (48.9)
Vancomycin	153/314 (48.7)
Trimethoprim/sulfamethoxazole	67/314 (21.3)
Clindamycin	59/314 (18.8)

Abbreviations: MRSA, methicillin-resistant *S. aureus*; MSSA, methicillin-susceptible *S. aureus*; SSTI, skin and soft tissue infection.

^a^In CHROME, receipt of at least 1 systemic antibiotic (nonoritavancin) related to the index infection within 30 days before the first dose of oritavancin.

^b^Cephalosporins included the following, in no specific order of frequency: ceftriaxone, cefepime/ceftazidime, cefazolin, ceftaroline, cephalexin/cefadroxil, cefuroxime, cefpodoxime.

Cultures of infected tissue or fluid demonstrated Gram-positive pathogens at baseline in 146 (33.2%) patients. Only 8 Gram-negative isolates were recovered, and all represented polymicrobial infections with either staphylococci or streptococci. *Staphylococcus aureus* was the predominant pathogen (74.0% of 146 patients with a definitive culture for Gram-positive bacteria), and the distribution revealed a predominance of MRSA over MSSA (59.3% vs 40.7%). Positive cultures recovered from 32 multiple-dose patients did not represent those for single-dose less complicated infections; however, *S. aureus* (MRSA, 9; MSSA, 6) remained the predominant pathogen isolated (63%, 15/24) (Table 5).

**Table 5.** Patients Treated With Multiple-Dose Oritavancin for Complicated Gram-Positive Infections

Age/ Sex	Infection	Pathogen(s)	Oritavancin Dosing	Site(s) of Infusion	Clinical Outcome	Adverse Events	Notes and Prior Therapy Outcomes
Osteomyelitis							
46/M	Osteomyelitis	MSSA, *S. pyogenes*	1200 mg × 6 every 6–8 d	HOIC	Cure	None	Prior amoxicillin therapy failure
47/F	Osteomyelitis	MRSA	1200 mg × 2 every 9 d	POIC	Failure	None	No prior therapy; ORI failure with change to doxycycline; eventual amputation
70/F	Osteomyelitis, chronic	MRSA	1200 mg × 10 every 7–8 d	OP-HOU	Improvement	None	No prior therapy
70/M	Osteomyelitis, due to traumatic wound	*S. pyogenes*	1200 mg × 2 every 6 d	HOIC	Cure	None	Prior cephalexin failure, changed to DAL with AE necessitating change to ORI
55/F	Osteomyelitis	Culture-negative	1200 mg × 3 every 14 d (with oral TMP/SMX)	POIC	Cure	None	Prior cefazolin. daptomycin, linezolid, PTZ, and VAN failures
36/F	Osteomyelitis, right ankle	MSSA	1200 mg × 2 every 14 d	POIC	Cure	None	Prior A/S, cefazolin, cephalexin, VAN, CFTX, and TMP/SMX failures
58/M	Osteomyelitis, left foot	MRSA	1200 mg × 1, then partial dose in 14 d	POIC	Improvement	Infusion-related reaction; sent to ED	Prior minocycline, VAN failure; change to linezolid following ORI AE
46/F	Osteomyelitis, chronic; skull	MRSA	1200 mg × 6 every 7–14 d	ED	Cure	None	Prior VAN therapy with improvement; patient requested hospital discharge and OPAT
Other Bone and Joint Infections							
43/F	Native septic arthritis/synovitis	Culture-negative	1200 mg × 5 every 6–14 d	OP-HOU	Improvement	None	No prior therapy
22/M	Septic arthritis/synovitis	*Bacillus* sp.	1200 mg × 2 every 14 d	IP, then ED	Cure	None	Prior cefazolin failure followed by VAN improvement
78/M	Prosthetic joint	Not cultured	1200 mg × 2 every 14 d	HOIC	Cure	None	Prior TMP/SMX with improvement
Skin and Soft Tissue Infections: Cellulitis							
74/F	Cellulitis, nonpurulent	MRSA	1200 mg × 2 every 11 d	POIC	Cure	None	Prior doxycycline with improvement
86/F	Cellulitis, nonpurulent	MSSA	1200 mg × 2 every 14 d	POIC	Improvement	None	No prior therapy
75/M	Cellulitis, nonpurulent	Not cultured	1200 mg × 2 every 14 d	POIC	Cure	None	No prior therapy
60/F	Cellulitis, purulent due to surgical wound	Coagulase-negative *Staphylococcus*	1200 mg × 4 every 7–17 d	OP-HOU	Improvement	None	Prior cephalexin and VAN failures; VAN therapy with AE and change to ORI
67/F	Cellulitis, purulent	*S. pyogenes*	1200 mg × 6 every 7–8 d	HOIC	Cure	None	Prior cefadroxil therapy failure
82/M	Cellulitis, nonpurulent	Not cultured	1200 mg × 2 every 13 d	POIC	Improvement	None	Prior PTZ and VAN therapy failure
50/F	Cellulitis, nonpurulent	Not cultured	1200 mg × 2 every 14 d	POIC	Cure	None	No prior therapy
48/M	Cellulitis, nonpurulent	Not cultured	1200 mg × 2 every 14 d	POIC	Cure	None	Prior telavancin with improvement
86/F	Cellulitis, nonpurulent	MRSA	1200 mg × 2 every 14 d	POIC	Cure	None	No prior therapy
60/M	Cellulitis, nonpurulent	Not cultured	1200 mg × 2 every 10 d	HOIC	Improvement	None	No prior therapy
Skin and Soft Tissue Infections: Abscess, Wound, Burn							
46/F	Abscess	MRSA	1200 mg × 2 every 14 d	HOIC	Cure	None	Prior tedizolid with improvement; early relapse necessitating ORI therapy
78/F	Abscess, surgical wound	MRSA	1200 mg × 2 every 14 d	POIC	Cure	None	Prior VAN therapy with improvement
60/M	Surgical wound	*E. faecalis*	1200 mg × 2 every 14 d	HOIC	Improvement	None	Prior amoxicillin and TMP/SMX failure
57/M	Surgical wound	*Corynebacterium* sp.	1200 mg × 6 every 6–8 d	OP-HOU	Improvement	None	No prior therapy
60/F	Surgical wound	Not cultured	1200 mg × 2 every 11 d	IP, then HH	Cure	None	No prior therapy
31/M	Surgical wound	MSSA	1200 mg × 2 every 14 d	POIC	Cure	None	Prior ceftazidime, MTZ, plus VAN therapy changed to ORI for MSSA culture result
67/M	Unspecified wounds	Coagulase-negative *Staphylococcus*	1200 mg × 7 every 6–8 d	OP-HOU	Cure	None	Prior amoxicillin/clavulanate failure
66/M	Wound, unspecified	MSSA	1200 mg × 2 every 8 d	OP-HOU	Cure	None	Prior TMP/SMX failure
56/M	Traumatic wound	*Corynebacterium striatum*	1200 mg × 3 every 14 d	OP-HOU	Failure	None	No prior therapy
24/M	Surgical wound, brain abscess	MRSA, MSSA	1200 mg × 9 every 6–7 d	ED	Cure	Mild nausea	Prior CFTX, clinda, nafcillin, VAN failure
51/M	Infected burn	Not cultured	1200 mg × 2 every 7 d	HOIC	Improvement	None	Prior cefepime plus VAN therapy improvement; limb amputation still required

Abbreviations: A/S, ampicillin-sulbactam; AE, adverse event; CFTX, ceftriaxone; DAL, dalbavancin; ED, emergency department; HH, home health; HOIC, hospital-owned infusion center; IP, inpatient; MRSA, methicillin-resistant *S. aureus*; MSSA, methicillin-susceptible *S. aureus*; MTZ, metronidazole; OP-HOU, outpatient hospital observation unit; ORI, oritavancin; POIC, physician-owned infusion center; PTZ, piperacillin-tazobactam; TMP/SMX, trimethoprim-sulfamethoxazole; VAN, vancomycin.

### Oritavancin Treatment Characteristics

Infection type was further examined in Table 3 according to the administration of single doses (408 patients, 92.7%) and multiple doses (32 patients, 7.3%) of oritavancin. Of 32 patients receiving multiple doses of oritavancin, as defined previously, 21 were for skin and soft tissue infections, and the remainder were for osteomyelitis (n = 8), septic arthritis/synovitis (n = 2), and prosthetic joint infection (n = 1). The use of oritavancin for complicated infections, listed as “other” in Tables 2 and 3, included 18 patients with osteomyelitis. Miscellaneous infections treated with single doses of oritavancin included catheter exit site (n = 1), infected bursa (n = 3), maxillary sinusitis (n = 1), lumbar wound (n = 1), and lymphadenitis (n = 1).

**Table 3.** Major Infection Classification and Dosing of Oritavancin, Single- and Multiple-Dose Oritavancin Regimens^a^

Infection Classification and Dosing	no./No. (%)
Skin and soft tissue	401/440 (91.1)
Single dose	380/401 (94.8)
Multiple dose	21/401 (5.2)
Bacteremia	7/440 (1.6)
Single	7/7 (100)
Multiple	0/7 (0)
Other	32/440 (7.3)
Osteomyelitis	18/32 (56.3)
Single dose	10/18 (55.6)
Multiple dose	8/18 (44.4)
Septic arthritis, synovitis	4/32 (12.5)
Single dose	2/4 (50.0)
Multiple dose	2/4 (50.0)
Prosthetic joint infection	3/32 (9.4)
Single dose	2/3 (66.7)
Multiple dose	1/3 (33.3)
Other^b^	7/32 (21.9)
Single dose	7/7 (100)
Multiple dose	0/7 (0)

^a^Multiple-dose oritavancin includes treatment courses in which doses were interrupted by no more than 14 days. For skin and soft tissue, multiple doses include cellulitis (n = 10), wound (n = 8), abscess (n = 2), and burn (n = 1).

^b^Includes catheter exit site, 1; infected bursa, 3; maxillary sinus infection, 1; hardware, posterior lumbar tissue, 1; and lymphadenitis, 1.

Investigators often incorporated oritavancin into treatment plans following prior antibiotic therapy to avoid daily intravenous infusions of alternative antibiotics. For example, in the 30 days before the first dose of oritavancin, 71.4% (314/440) of patients received at least 1 nonoritavancin systemic antimicrobial to treat a variety of Gram-positive infections. Cephalosporins (48.9%), vancomycin (48.7%), trimethoprim/sulfamethoxazole (21.3%), and clindamycin (18.8%) were the most common antibiotic classes used in treatment regimens in the pre-oritavancin period. In patients receiving >1 dose of oritavancin with doses no more than 14 days apart, only 1 patient received a systemic antibiotic as concomitant therapy for the documented Gram-positive pathogen. Other concomitant therapies in 8 additional patients were primarily for treatment of secondary Gram-negative infections. After the last dose of oritavancin, 44 patients received additional systemic antibiotics targeting Gram-positive pathogens; these included treatment failure in 31 of 52 patients (59.6%), adverse events to oritavancin in 2 patients, and prevention of recurrence in 11 patients.

### Clinical and Microbiologic Outcomes

Clinical outcomes are provided in Table 4 for receipt of single and multiple doses of oritavancin in 438 evaluable patients. Overall, clinical success (cure or improvement) was observed in 88.1% (386/438) of patients. Separately, clinical success rates of 87.7% (356/406) and 93.8% (30/32) were observed for patients receiving single-dose and multiple-dose (separated by no more than 14 days) regimens, respectively. In addition, clinical cure was reported for 64.4% (282/438), and clinical improvement was reported for 23.7% (104/438) of patients. Seven patients with bacteremia treated with single doses of oritavancin were clinical cures.

**Table 4.** Clinical and Microbiologic Outcomes in 438 Evaluable Patients^a^

Outcome	Single Dose, no./No. (%)	Multiple Doses (Interrupted by ≤14 d), no./No. (%)	Overall, no./No. (%)
Clinical success^b^	356/406 (87.7)	30/32 (93.8)	386/438 (88.1)
Clinical cure	262/406 (64.5)	21/32 (62.5)	282/438 (64.4)
Clinical improvement	94/406 (23.2)	10/32 (31.3)	104/438 (23.7)
Clinical failure	50/406 (12.3)	2/32 (6.2)	52/438 (11.9)
Microbiological eradication^c^	28/37 (75.7)	1/37 (2.7)	29/37 (78.4)
Microbiological persistence^c^	7/37 (18.9)	1/37 (2.7)	8/37 (21.6)

^a^Two patients had no clinical outcomes reported.

^b^Clinical success includes clinical cure and clinical improvement, as assessed within 30 days after oritavancin administration.

^c^Microbiologic assessment includes laboratory-confirmed microbial eradication or persistence of the same baseline pathogen at the site of the initial infection. One patient in the multiple-dose group (3 doses within 14 days) revealed microbiologic persistence, whereas 1 patient in the multiple-dose group (2 doses within 14 days) revealed microbiologic eradication.

Microbiologic response post-therapy was not performed in most patients; cultures were obtained in part to evaluate potential causes of clinical failure. In the 7 bacteremic patients, additional blood cultures were not obtained during or at end of therapy. Results are shown in Table 4. In 37 patients with post-therapy follow-up cultures, microbiologic eradication was achieved in 29 patients and microbiologic persistence was documented in 8 patients with various complicated skin and soft tissue infections; persistence of the baseline pathogen was associated with single oritavancin doses in 7 patients and multiple doses in 1 patient. Also, of 8 patients with persisting pathogens, 4 were assessed as clinical success and 4 were deemed clinical failures. These clinical successes were deemed to represent colonization that did not require treatment.

### Multiple Dose Oritavancin Use

Empirically, interruption between doses by 14 days or less (ie, interdose spacing) was the definition utilized to examine clinical and microbiologic outcomes in the cohort of patients who were administered at least 2 doses of oritavancin. This definition was met by 32 of 440 (7.3%) patients treated with 2–10 doses (mode, 2 doses; mean, 3.3 doses) of oritavancin (Table 5). Eight of 26 (31%) sites incorporated multiple-dose strategies to treat at least 1 patient. The first patient in this study with a multiple-dose oritavancin regimen was included in April 2016. Infections included bone and joint (n = 11, including 8 with osteomyelitis), cellulitis (n = 10), wound (n = 8), abscess (n = 2), and burn (n = 1). Multiple-dose therapies were administered in physician-owned infusion centers (n = 13), hospital-owned infusion centers (n = 8), outpatient hospital observation units (n = 7), and emergency departments (n = 3). Patient antibiotic therapies administered before oritavancin are listed in Table 5. Of these 32 patients, 10 received no prior nonoritavancin antibiotics, including 2 of the 8 osteomyelitis patients. Clinical success was observed in 30 of 32 patients (93.8%) overall, including 10 of 11 patients (90.9%) with bone and joint infections and 7 of 8 (87.5%) patients with osteomyelitis specifically. Clinical failures in 2 patients included 1 patient ultimately requiring amputation due to an MRSA-associated osteomyelitis and a second patient with an unresolved traumatic wound with a positive culture for *Corynebacterium striatum* that persisted after 3 doses of oritavancin infused every 14 days.

### Osteomyelitis and Joint Infection

CHROME included 18 patients treated with oritavancin for osteomyelitis (Tables 2 and 3). The majority of these patients (77.8%) received prior antibiotic therapy, of which 50% (9/18) were considered clinical failures before receiving oritavancin. Eight patients received multiple doses of oritavancin (range, 2–10 doses) for the treatment of osteomyelitis. An additional 10 patients received oritavancin as a single dose for completion of therapy. Clinical success (8 cures, 1 improvement) was observed in 9/10 (90.0%) of these single-dosed patients.

Seven patients received oritavancin for treatment of joint infections, including septic arthritis/synovitis (n = 4, 2 single-dosed and 2 multiple-dosed patients) and prosthetic joint infections (n = 3, 2 single-dosed patients and 1 multiple-dosed patient) (Tables 2 and 3). Four of 7 patients received prior antibiotic therapy, of whom 1 was considered a failure. Clinical success following oritavancin was observed in 5 of 7 patients (71.4%). Two patients were considered clinical failures; both received single doses. In 2 single-dose oritavancin patients with septic arthritis/synovitis, 1 patient failed due to a retained foreign body. In 2 single-dose patients with prosthetic joint infections, 1 patient was cured but the other failed oritavancin. Both of these patients suffered multiple prior antibiotic therapies including various beta-lactams, vancomycin, clindamycin, and/or daptomycin.

### Safety Outcomes

The evaluable safety cohort consisted of 440 patients who received either a single dose (n = 408) or multiple doses (n = 32) of oritavancin (Table 6). Oritavancin was safe and well tolerated. There were 6.6% (29/440) patients who experienced at least 1 potential treatment-emergent adverse event (TEAE). The most common event was pruritis (3.2%, 14/440). There was 1 patient (0.2%) who experienced 3 serious AEs (nausea, vomiting, and asthenia). Oritavancin was discontinued in 6 patients in response to a TEAE. These events included, by patient, (1) infusion site reaction; (2) pruritis, urticaria, and headache; (3) urticaria and pruritis; (4) headache and throat tightness; (5) nonspecified infusion-related reaction; and (6) back pain and flushing. One of the 6 patients experienced an infusion site reaction (second dose 14 days after the first dose) and was observed in the emergency room and discharged within a few hours. In the other 5 patients, adverse events resolved spontaneously within 90 minutes after discontinuation; all patients were discharged home. All AEs were manifested between 25 minutes and 2 hours from initiation of a 3-hour infusion. There were no deaths during the observation period.

**Table 6.** Treatment-Emergent Adverse Events in CHROME for Oritavancin-Treated Single-Dose and Multiple-Dose Patients^a^

Incidence of Selected Adverse Event			All CHROME Patients (n = 440), No. (%)
Hypersensitivity			5 (1.1)
Diarrhea			3 (0.7)
Vomiting			3 (0.7)
*Clostridioides difficile*–associated diarrhea^b^			1 (0.2)
Adverse Event	Single-Dose (n = 408), No. (%)	Multiple- Dose (n = 32), No. (%)	All CHROME Patients (n = 440), No. (%)
Patients with a drug-related adverse event	27 (6.6)	2 (6.3)	29 (6.6)
Patients with a drug-related serious adverse event	1 (0.2)	0 (0)	1 (0.2)
Discontinuation due to any adverse event	5 (1.2)	1 (3.1)	6 (1.4)

^a^Adverse events with a reasonable possibility of a causal relationship to oritavancin, as assessed by the investigator, were reported.

^b^ *Clostridium difficile*–associated diarrhea was identified in a single-dose patient.

## DISCUSSION

Observational studies in real-world settings can serve as useful complements to rigorous randomized clinical trials (RCTs). Such observational studies have the advantage of studying patients who are usually excluded from RCTs while acknowledging the use of antibiotics and their dosing, which reflects actual use in clinical practice. This descriptive analysis pools the results from an earlier published study [17]. The supplemental 328 patients acquired since then result in a 440-patient pooled analysis, presented in this report.

The pivotal trials conducted for oritavancin included only single-dose regimens for ABSSSI and excluded patients with complicated infections such as osteomyelitis. Several recent patient cases and case series describe the experiential real-world use of oritavancin in multiple-dose regimens for the treatment of bone and joint infections, pneumonia, bacteremia, and complicated surgical site infections [9–16]. In this study, we sought to build upon these prior reports of real-world oritavancin use by describing the clinical characteristics and outcomes of a diverse cohort of patients with Gram-positive infections treated with at least 1 dose of oritavancin.

This retrospective, observational study (CHROME) characterizes the clinical outcomes and adverse events of adult patients who received at least 1 dose of oritavancin for the treatment of acute bacterial skin and skin structure infections and complicated infections caused by Gram-positive pathogens, such as osteomyelitis. Overall, clinical success in evaluable patients receiving at least 1 dose of oritavancin was 88.1% (386/438). Clinical success rates were similar between patients in the single-dose cohort (87.7%, 356/406) and those in the multiple-dose cohort (93.8%, 30/32) and reflect that observed in the pooled SOLO clinical trials for oritavancin (92.6%, 760/821) [3–5]. A drug-related adverse event was found in 6.6% (29/440) of patients. Incidence of serious AEs and discontinuation of oritavancin infusions were low in CHROME, similar to the SOLO trials [18]. Therefore, CHROME provides further evidence of the safety and tolerability of oritavancin, which was previously demonstrated in the SOLO trials, and confirms that oritavancin is an effective and well-tolerated long-acting lipoglycopeptide antibiotic used as single-dose treatment of ABSSSI, its approved indication, but also as a multiple-dose regimen for treatment of complicated Gram-positive infections. In this study, all oritavancin doses were 1200 mg infused over 3 hours; this was the practice at enrolling sites.

Specifically, CHROME also demonstrates the use of oritavancin in complicated Gram-positive infections, including osteomyelitis. A recent retrospective study by Schulz and colleagues [12] reported 17 patients who received multiple oritavancin doses (range, 2–18 doses) for the treatment of complicated Gram-positive infections including osteomyelitis in 4 patients, as well as patients with surgical site infection, intravascular infections, and pneumonia. All patients achieved clinical response with oritavancin. Four patients (24%) had an adverse event that reversed rapidly after discontinuation of oritavancin. Although Schulz and colleagues did not obtain plasma concentrations, others have shown that trough levels of oritavancin remained low and were similar to those reported in the literature from prior clinical studies with single-dose oritavancin [2, 10, 14]. A 9-patient series by Chastain and Davis treated lower-extremity chronic osteomyelitis with 2 or more doses of oritavancin and documented clinical success in all patients, without any drug-related adverse events [15]. An additional case of hardware-associated vertebral osteomyelitis due to vancomycin-resistant and daptomycin-nonsusceptible *Enterococcus faecium* responded with clinical improvement to a 10-dose course of oritavancin plus continuous infusion ampicillin (12 g/d); magnetic resonance imaging at the end of therapy revealed a >90% reduction in the size of the fluid collection [16]. Stewart and colleagues [11] reported 10 patients with invasive Gram-positive infections, although only 1 received multiple doses of oritavancin—a 26-year-old female with sacral joint osteomyelitis. Delaportas and colleagues [13] reported on a 49-year-old female with right tibial osteomyelitis secondary to a retained intramedullary surgical pin who underwent a course of 6 weekly doses for MSSA osteomyelitis. Antony and Cooper [9] presented 2 patients with prosthetic joint infections who were treated successfully with single-stage revisions, antibiotic spacers, and 2–4 doses of oritavancin 1200 mg infused over 3 hours, spaced 10 days apart. Finally, Foster and colleagues [14] presented 1 case of a 6-weekly dose treatment course with oritavancin of femoral osteomyelitis due to vancomycin-resistant *Enterococcus faecium*. The contributions of surgical debridement, patency of local vasculature, and multiple prior nonoritavancin therapies to clinical outcomes in these patients are difficult to assess.

This study has important limitations. Data collected during this study include the retrospective, noncomparative, unblinded, and nonrandomized nature of the real-world evidence. Assessment of efficacy was based on subjective assessment extracted from the medical record by the investigators. Missing data may have been encountered given the 30-day clinical assessment and 60-day safety evaluation windows. Although quality data checks were performed, the results of this study pertaining to patients receiving multiple-dose regimens of oritavancin should be verified in larger multicenter open-label cohort studies enrolling patients with complicated and microbiologically documented infections. Finally, >90% of patients included in this study were identified as white. Therefore, additional data should be sought to validate the findings of this study in nonwhite patients.

## CONCLUSIONS

Use of both single-dose and multiple-dose regimens of oritavancin for the treatment of ABSSSIs and other infections may be an effective and safe alternative to daily infusions of shorter half-life antibiotics. In addition, these therapeutic strategies may be especially safe and effective alternatives to daily antibiotic infusions to facilitate treatment of ABSSSIs and more complicated infectious disease processes in the outpatient setting [15–17]. CHROME provided information on oritavancin real-world use in 440 patients, many with complex Gram-positive infections including osteomyelitis. The observation that oritavancin was preferentially administered to patients with complicated Gram-positive infections mostly in the outpatient setting, including 32 patients who received scheduled weekly or every-2-week oritavancin doses, reflects a unique option for many patient management cases. Oritavancin appears at this juncture to be a safe and effective alternative to daily antibiotic infusions to treat complicated Gram-positive infectious disease processes.
